# Supplementary material for: Evaluating a large language model’s ability to solve programming exercises from an introductory bioinformatics course
Source: PLoS Comput Biol. 2023 Sep 28;19(9):e1011511. doi: 10.1371/journal.pcbi.1011511 (PMC10564134; doi:10.1371/journal.pcbi.1011511)
Supplement: Attachment — Submitted filename: PLOS_Comp_Bio_Response_to_Reviewers.pdf [file pcbi.1011511.s001.pdf]

DEPARTMENT OF BIOLOGY

Dear Dr. Ouellette:

Thank you for taking time to be the editor for our manuscript. We have carefully addressed the reviewers' comments. Below we provide a point-by-point response. The review comments are in light gray. Our responses are in black and bolded (so they are easy to see). We have also edited other parts of the text for clarity.

Please let me know if we can clarify anything.

Warm regards,

Stephen R. Piccolo, PhD
Associate Professor
Department of Biology
Brigham Young University
(801) 422-7116
Stephen_Piccolo@byu.edu

Reviewer #1: This manuscript argues that chatGPT can and should be used in research settings in bioinformatics, and, to a lesser extent, in teaching coding. The work is based on the experience of the authors in teaching an introductory coding class for bioinformatics, using code generated by querying ChatGPT with problems prepared for that class, and investigating the solutions provided via an automated grader used in educational settings.

The exercises the authors provide seem to be very basic introductory programming tasks for teaching basic coding concepts. String handling, control structures, loops, lists, dictionaries, etc. Some are bioinformatics-adapted and the final tasks seem to be more advanced with use of Pandas, CLI parsing and some visualization. However, those more complex tasks, by the authors own data, are the tasks in which ChatGPT failed. Therefore, I do not see evidence that ChatGPT I believe that the title, “Many bioinformatics programming tasks can be automated with ChatGPT”, is misleading, and should perhaps be changed to “ChatGPT receives a C grade on introductory programming tasks”.

Thank you for taking time to review the manuscript. We can see that a more nuanced approach to interpreting the results is in order, and we have made several changes along these lines. For starters, we have changed the title to "Evaluating a large language model's ability to solve programming exercises from an introductory bioinformatics course." The reviewer suggested that the title should reflect ChatGPT's performance after one attempt

(75.5% success rate). However, we were deliberate in our choice to allow ChatGPT up to 10 attempts to solve each exercise. Firstly, this criterion aligns with our pedagogical approach. In our course, the exercises are *formative*. Accordingly, failing, receiving feedback, and then re-attempting are part of the learning process, as reviewed in *Shute VJ. Focus on formative feedback. Review of educational research. 2008 Mar;78(1):153-89*. Secondly, allowing multiple attempts can be justified in relation to how biologists could use LLMs in research. A key to this assertion is that researchers must verify code for functional correctness (as the reviewer suggests). Iteratively seeking help and checking for functional correctness is important when receiving help from *any* source. In this experiment, we provided basic functional tests; however, in a real-world setting, a researcher would need to create functional tests. If LLM-generated code does not function correctly on the first attempt, the researcher could ask the model to revise the solution or generate a new solution. In our experience, performing this iterative process for up to 10 iterations would take a matter of minutes (assuming functional tests have been created). When we allowed up to 10 attempts per exercise, ChatGPT solved 97.3% of the exercises, including all but 2 of the tasks that involved the use of Pandas, CLI parsing, or visualization. Thirdly, allowing multiple attempts per exercise is consistent with what others have reported in the literature (for example, Austin, et al., Finnie-Ansley, et al.). We have updated the Abstract and the Discussion section to provide clarification on these points.

Having read this manuscript, I believe it clearly demonstrates why using ChatGPT as a programming aide for scientists is harmful, and why teaching programming with ChatGPT is probably even more harmful. To do so I will start by quoting the first sentence in the Discussion:

"These findings are remarkable and signal a new era for life scientists. For many basic- to moderate-level programming tasks, researchers no longer need to write code from scratch."

We have modified this statement (and elsewhere in the manuscript) to provide more nuance about LLMs' potential usefulness in education and research settings. In addition, we emphasize that careful functional validation of LLM-generated code remains critical.

First, I will address this statement that has to do with the suggested use of ChatGPT in scientific coding. I see this statement as highly problematic. First: the authors have shown that for 25% of the tasks, ChatGPT requires several iterations until it generates a correct answer, if ever. Students are expected to have a higher success rate when submitting their exercise. One may argue that the back-and-forthing the authors did with ChatGPT to improve the code might be equivalent to the debugging process prior to grade submission, but it isn't.

As described in our earlier comments, we believe that iteratively generating code and receiving feedback on functional correctness is a valid approach both in an educational setting and in research settings. Without approval we cannot provide details; but we can say that a 75% success rate on the first attempt is higher than many students in our classes. However, we do not argue that our interactions with ChatGPT are equivalent to how a researcher would do so.

Future work might enable us to better understand how/whether instructors can use LLMs to facilitate better learning.

Second, the authors seem to advocate the use of ChatGPT to simplify simple code writing, pretty much the same way a calculator is used to simplify arithmetic: a tool to avoid basic errors, and to get on with the more important tasks of using the code for analysis rather than investing time in writing and debugging the code. Except that a calculator is always right (2+3=5, always) whereas with ChatGPT the answer is 25% wrong as it might vary between 2+3=4 in one iteration and 2+3=6 in another, with various other plausible but wrong results. Relying on ChatGPT-generated code would be like relying on a calculator that generated only 75% of the answers correctly in the first try. That scientist will not have the capability to debug the code. A coding-competent scientist may be tempted to use ChatGPT to write the code, and gloss over the details of the result. If one sees ChatGPT as a standard of truth, without understanding the code at all, one does not have the necessary skills to debug. Debugging, and not initial code writing, is the bulk of programming time, effort, and ability as coding is mostly about debugging and further adapting code to changing libraries, operating system, language versions and user input and requirements. The latter is especially true for research code which is used for exploratory analysis. If one does not have the skills to debug, one will not have the skills to modify code as required when the analysis requires changing, when the format of the input data changes, or any other myriad issues. They will have no tools for creating software tests that look for things such as edge cases and other problems that may arise with ChatGPT-generated code that is plausible, yet will fail, and the scientist generating the code will not even know that it failed, as the output will be plausible, but wrong. In a good scenario, the code generated by ChatGPT may not be completely wrong, and provide correct analysis to input, but only in some cases.

We agree with most of what the reviewer stated here. We do not advocate for blindly using the outputs of LLMs. ChatGPT and other LLMs often produce code that does not meet stated requirements (and/or edge cases that may not have been specified as part of the prompts). As a (simple) means of validation, we compared ChatGPT's output against the expected output that we had defined before beginning the study. This approach aligns with the educational context that we considered (an introductory course). However, in subsequent courses and in the "real world," other types of validation would be critical. Learning to perform functional validation, debug code, and "[adapt] code to changing libraries, operating system, language versions and user input and requirements" are also critical skills for scientists to learn. We have updated the Discussion to be more clear on these points. However, we maintain that these needs do not negate the potential for LLMs to be useful (when used appropriately) to students and researchers.

Finally, I don’t see any of the tasks they provided to ChatGPT as “moderate”. They are all introductory and easy. Most have little real-world relevance, although they have great educational value in teaching basic coding.

Thank you. In our original submission, we used "moderate" to refer to exercises that come toward the end of the course. In our view, some of these exercises are more difficult than the classical "rainfall" problem, which has been considered for decades to be a barometer

for students' programming abilities at the end of their first course (*Seppälä, Otto, et al. "Do we know how difficult the rainfall problem is?." Proceedings of the 15th Koli Calling Conference on Computing Education Research. 2015.*) However, we do *not* want to imply that these exercises reflect the skill level of a moderately experienced bioinformatician. Accordingly, we have updated the title and adjusted wording throughout the manuscript to avoid this confusion. For example, in the Discussion section, we state that "The programming exercises we evaluated do not necessarily represent skills…that would be used broadly in bioinformatics research."

As for the educational aspect: here the authors seem to be more reserved. Quote: "Our findings have important implications for education. Until LLMs demonstrate an ability to replace all human programming efforts, it will remain necessary for students (and others) to gain programming skills". This statement has the implicit assumption that programming skills have a threshold level that needs to be acquired. I disagree with that assumption, as programming is an iterative process that requires domain knowledge, creativity, synthesis, and many other skills. There is no threshold, there is constant skill acquisition in many dimensions.

We meant to speculate that perhaps, at some point, LLMs will completely obviate the need for programming skills. But as the reviewer implies, this may never happen, and other related skills will likely always be necessary. We have updated the Discussion to clarify this point.

However, I do agree with the authors that programming skills should be gained and that students should be able to do them themselves without resorting to a code generating LLM. To go back to the calculator analogy, one needs to know how to use a pencil-and-paper addition or long division, even if one will hardly ever use it in the future, otherwise one is arithmetically illiterate. The auction they advocate here is well-placed. It is therefore important to educate programming without the use of an LLM, as the authors suggest.

Thank you.

To go to the Conclusions: "In this study, we provide evidence that dialogue-based LLMs, such as ChatGPT, can aid in solving programming exercises in the life sciences, particularly in the field of bioinformatics." I disagree. The authors have not demonstrated that they have provided anything but very basic problems to chatGPT, and I am not sure that they are research or real-world-relevant bioinformatics tasks but rather very basic programming exercises.

We have modified this wording to clarify that these were basic programming exercises and to not imply that these exercises represent real-world bioinformatics tasks.

"However, despite generally excellent performance, these models cannot replace the need for human programming efforts entirely."

We have modified this wording to clarify that much remains to be learned about the extent to which these models can replace human programming efforts.

75% is not “excellent”, it is a “C” grade. Especially on the very simple problems that the authors placed in the Additional Materials.

Please see our earlier comments about our reasoning for allowing the LLM to make multiple attempts per exercise.

“In an authentic research setting, where an auto-grader is not available to provide instant feedback on the correctness of model-generated code, there is a risk that relying on their outputs may produce erroneous results.” I agree. And it may be harmful.

Thank you.

“With the help of machine learning models, instructors can provide more personalized and efficient feedback to students, and researchers can accelerate their work by automating programming tasks”

There is no demonstration of the former regarding students (how will instructors provide more personalized and efficient feedback to students using an LLM rather than grading software? The LLM in question was used for task solving, not for task feedback). As for the latter, using a code generator that is 25% wrong on even the most simple tasks (which are not even applicable in an authentic research or other real-world setting), does not accelerate research. I suspect that it probably slows it down and introduces unnecessary errors.

Our statements were speculative. More research is needed on this topic. We have updated the wording in this section to clarify this.

My suggestion is for the authors to review the data and their conclusions. It seems to me that the results clearly demonstrate that the use of an LLM for generating basic code, and worse, for teaching programming, should be highly discouraged.

We appreciate that readers will have differing viewpoints on these topics after reviewing our study.

Minor comments:
Please provide the URL for the OBF repository in the Data Availability Section

We have moved everything to a GitHub repository and now provide the URL in the manuscript.

Please provide Results.html also as an Excel sheet or an ods file. It is hard to read as HTML.

Thank you for this suggestion. We attempted to use Excel, but the number of characters in some of the programming prompts exceeded the maximum limit for a cell, so the prompts were truncated. Thus, we used HTML. Although the HTML solution has disadvantages, we

feel it is a reasonable compromise. The GitHub repository provides code demonstrating how to read the HTML file into a data frame in R. Other researchers can follow this example to export the data to other formats. Our manuscript now provides clarification on this point.

Which Version of ChatGPT (dated, if possible) and Python were used?

We have clarified these points in the manuscript.

Reviewer #2: The manuscript from Piccolo, Denny, Luxton-Reilly, Payne and Ridge explains the significance of large language models like ChatGPT applied to bioinformatics programming tasks. The methods explain how the researchers tested ChatGPT on several tasks, and evaluated its performance and the ability to correct answers guided by human prompts. The researchers found that ChatGPT solved 97.3% of the tasks within 7 or fewer attempts.
I believe the study to be very timely and interesting, but I have some suggestions on how to clarify its purpose:

Thank you for taking time to review the manuscript.

- I would change the title to be less specific to ChatGPT and more general about LLMs. I understand that ChatGPT is well-known and highly discussed at the moment, and that this is the LLM that was used for the study, however I believe that the results of the study are applicable beyond ChatGPT only, and that keeping it in the title exposes the manuscript to the danger of "early obsolescence". This is just a suggestion and I would leave the decision to the authors preference, also considering their sentence in the discussion about the limitations of the study applicability.

Thank you for this suggestion. We have updated the title. The new title does not specifically mention ChatGPT.

- In the introduction, I believe that the purpose of the study should be better clarified. Was it to test if bioinformaticians' job can be sped up? Do the authors think they could quantify this, comparing the time to formulate ChatGPT prompts and to write code independently?
- Or was the purpose to discuss ChatGPT's potential as a learning tool? The introduction mentions that "learning to
program is a daunting challenge for many researchers". To discuss LLMs' implications in this field, I believe more data should be provided about the level of experience of the humans providing prompts to ChatGPT in this study. Were they all experts, even though they "took the stance of a naive programmer"? If so, would ChatGPT performance result change if the prompts were formulated by real beginner programmers?

Thank you for these questions. We have updated the 4th paragraph in the Introduction to clarify this purpose.

When discussing implications, we considered whether ChatGPT might be useful as a learning tool or to make researchers more efficient. However, with the study design that we used, it would be infeasible to quantify whether a bioinformatian's work can be accelerated with LLMs.

We have provided clarification about the level of experience of the human providing prompts to the models.

We now provide examples that suggest how students might interact with the models differently than the instructor did.

- Even though all the conversations between humans and ChatGPT are shared, I would appreciate more summarizing considerations about the prompts to correct ChatGPT's previous errors. I don't find the explanation exhaustive: "our feedback focused on helping ChatGPT understand the exercise requirements and was restricted to natural language such that no source code was present in any of our prompts". Was the type of error (generated by the previous version of the code) provided? If not, and if it was rather an effort to rephrasing the prompt in such a way that it could better understood by the LLM, how much expert knowledge was involved in this process? And how did the researchers decide between the two alternatives: "helping ChatGPT understand the exercise" and "indicated that the previously generated solutions were unsuccessful and asked ChatGPT to try an alternative approach"?

We have reviewed all interactions between the user and ChatGPT and provided a summary of these interactions (Table 1). Additionally, we have added text on this point to the Discussion.

I believe that the additional details suggested above would strengthen the message of the paper. In addition, the ambiguity I find in the introduction (is the purpose to demonstrate ChatGPT efficacy in learning or in working tasks?) reflects in some conflicts I find in the final discussion.
- If the purpose is to demonstrate that bioinformaticians will work quicker with ChatGPT, if we assume that they will all do once they get a job in the field, and if the purpose of the course is to prepare them to their future working environments, why summative assessments at the University do not allow access to the Internet?

This is an important question and one that many educators grapple with. One could argue that on summative assessments, students should have access to all of the resources that they would have in the "real world." The same could be argued for fact-based or math-based assessments. Some educators believe that it is important for students to demonstrate mastery independently. Other educators focus more on teaching students to use outside resources. For this introductory course, we currently use the former approach.

- In relation to the above, the manuscript comments on the danger of "over-reliance by novices" and this suggests that this is the reason why summative assessment still don't incorporate the use of LLMs. Hence, the discussion seems to indicate that LLMs are not considered by the authors

(yet) a good tool for learning, but this is not what suggested by the introduction (e.g. "learning to program is a daunting challenge for many researchers").

The data show that LLMs are effective at solving introductory bioinformatics programming exercises. We can only speculate about whether they are effective for helping students to learn. Answering this question will require further empirical research. We have clarified this in the Discussion section.

- I would also be interested in knowing more about the suggestions "one way that instructors could counter this behavior is to use LLMs to generate student-specific questions about their code" and "when students attempt to devise solutions but become stuck, they may be able to use LLMs as intelligent tutors".

Thank you for your interest in these topics. We have expanded this part of the Discussion to provide more clarity.

In summary, I believe the study to be relevant, well structured and deserving to be published. However, I would appreciate a better clarity on its premises, aims, and conclusions from the point of view of the authors. Giving the inherently subjective nature of my comments, I am anyway happy for the authors to incorporate my suggestions to the extent they see fit and I would accept the submission even with minor or no changes.

Thank you.

Reviewer #3: The work tests the use of LLM, chatGPT specifically to generate bioinformatics relevant programming code. The authors conclude that 1)the chatGPT solutions are generic and can fail in providing correct solutions for specific biology problems 2) human in the loop is required to evaluate the solutions 3) since the LLM models are easily accessible the educators will have to account for their usage by themselves and by their students and adapt teaching and assessments accordingly.

It would be good to provide a clear table of DOS and DONTS

Thank you for this suggestion. We now provide a list of do's and don'ts (Figure 6).

The versions of GPT model used must be mentioned

We have added this information.

A big gap in the study is to show comparison with another LLM model - it is likely that the responses will vary between models and it is important to highlight those differences due to prompt, model types and model training.

We have added a comparison against Google Bard for approximately one third of the programming exercises (see Methods). We believe that a more comprehensive comparison

should be saved for subsequent work and should include many different LLMs. Our results suggest that Bard is less effective than GPT at solving these exercises but often does quite well.

While LLM models can be used in assisting with coding tasks, it does require a human in the loop to evaluate the outcomes. The current solutions are not self sufficient.

We agree and now address this point in the revised manuscript.

Have the authors made all data and (if applicable) computational code underlying the findings in their manuscript fully available?

The PLOS Data policy requires authors to make all data and code underlying the findings described in their manuscript fully available without restriction, with rare exception (please refer to the Data Availability Statement in the manuscript PDF file). The data and code should be provided as part of the manuscript or its supporting information, or deposited to a public repository. For example, in addition to summary statistics, the data points behind means, medians and variance measures should be available. If there are restrictions on publicly sharing data or code —e.g. participant privacy or use of data from a third party—those must be specified.

Reviewer #1: Yes

Reviewer #2: Yes

Reviewer #3: Yes

PLOS authors have the option to publish the peer review history of their article (what does this mean?). If published, this will include your full peer review and any attached files.

If you choose “no”, your identity will remain anonymous but your review may still be made public.

Do you want your identity to be public for this peer review? For information about this choice, including consent withdrawal, please see our Privacy Policy.

Reviewer #1: No

Reviewer #2: Yes: Lisanna Paladin

Reviewer #3: No

Figure Files:

While revising your submission, please upload your figure files to the Preflight Analysis and Conversion Engine (PACE) digital diagnostic tool, https://pacev2.apexcovantage.com. PACE

helps ensure that figures meet PLOS requirements. To use PACE, you must first register as a user. Then, login and navigate to the UPLOAD tab, where you will find detailed instructions on how to use the tool. If you encounter any issues or have any questions when using PACE, please email us at figures@plos.org.

Done

Data Requirements:

Please note that, as a condition of publication, PLOS' data policy requires that you make available all data used to draw the conclusions outlined in your manuscript. Data must be deposited in an appropriate repository, included within the body of the manuscript, or uploaded as supporting information. This includes all numerical values that were used to generate graphs, histograms etc.. For an example in PLOS Biology see here: http://www.plosbiology.org/article/info%3Adoi%2F10.1371%2Fjournal.pbio.1001908#s5.

Done
